# Total parathyroidectomy with forearm auto-transplantation improves the quality of life and reduces the recurrence of secondary hyperparathyroidism in chronic kidney disease patients

**DOI:** 10.1097/MD.0000000000009050

**Published:** 2017-12-08

**Authors:** Jia-Gen Li, Zhang-Sheng Xiao, Xian-Jie Hu, Yun Li, Xing Zhang, Song-Ze Zhang, Ai-Qin Shan

**Affiliations:** a Department of Thyroid Breast Surgery; b Department of Nephrology, Yinzhou Hospital of Ningbo University Medical College, Ningbo, China.

**Keywords:** forearm autotransplantation, quality of life, recurrence, secondary hyperparathyroidism, total parathyroidectomy

## Abstract

**Background:**

Our study aims to explore the effect of total parathyroidectomy (PTX) with forearm autotransplantation (FAT) on the quality of life and recurrence of secondary hyperparathyroidism (SHPT) in chronic kidney disease patients.

**Methods:**

A total of 104 chronic kidney disease patients with SHPT were enrolled and divided into the PTX (n = 62) and PTX + FAT (n = 42) groups. The operation efficacy was evaluated by analyzing preoperative and postoperative values, including levels of intact parathyroid hormone (iPTH), serum phosphorus, serum calcium, alkaline phosphatase (ALP), calcium-phosphorus product, signs and symptoms, and MOS 36-item short-form health survey (SF-36) scores. Moreover, complications and recurrences were followed up for 12 months after the operation. Binary logistic regression was to present the risk factors for the recurrence of chronic kidney disease patients with SHPT.

**Results:**

Compared with the preoperative values, the PTX and PTX + FAT groups showed decrease postoperative levels of iPTH, serum phosphorus, serum calcium, calcium-phosphorus product, bone pain, and skin pruritus at all time periods. The PTX and PTX + FAT groups demonstrated decreased ALP, fracture or deformity, and coronary artery calcification at 1 month, decreased short stature at 3 months after the operation but increased SF-36 score after operation. Compared with the PTX group, the level of iPTH decreased and the levels of serum calcium, calcium-phosphorus product increased at 3, 6, and 12 months after the operation in the PTX + FAT group. The levels of ALP, fracture or deformity, short stature, and SF-36 decreased separately at 1 week and 6 and 12 months after the operation, along with the decrease of coronary artery calcification and the recurrence rate, respectively, at 6 and 12 months after the operation in the PTX + FAT group when compared with those in the PTX group. Logistic regression analysis evidenced that the preoperative iPTH level, SF-36 score, and operation type were the risk factors for the recurrence of chronic kidney disease with SHPT.

**Conclusion:**

Total PTX combined with FAT is more effective in improving the quality of life and reducing the recurrence of chronic kidney disease with SHPT than PTX alone.

## 1 Introduction

Secondary hyperparathyroidism (SHPT) is a common severe complication in patients with end-stage renal disease (ESRD), and often persists after successful renal transplantation.^[1]^ SHPT is characterized by an increase in the parathyroid hormone (PTH) synthesis and secretion and hyperplasia of progressive parathyroid gland.^[2]^ Fundamentally, the etiology of SHPT is known as long-term parathyroid hyperplasia, which results in the formation of functionally independent adenoma.^[3]^ SHPT in patients can lead to high-turnover bone disease, interstitial and vascular calcifications, and as well as cardiovascular mortality and morbidity.^[4–7]^ The diagnosis of SHPT in patients mainly relies on high-resolution ultrasonography with color Doppler imaging (US/CD) and 99 mTc-methoxyisobutylisonitrile (MIBI) scintigraphy.^[8]^ Owing to the high occurrence and recurrence of SHPT, the patients’ quality of life is seriously affected.^[9]^ Therefore, multiple efforts have been made for the search of better solutions for SHPT.

Cinacalcet and Vitamin D are the commonly used drugs, and they are used in combinations to treat SHPT in patients on dialysis.^[10]^ Although conventional drug treatment has improved a lot in recent years, the parathyroidectomy demand still remains high, particularly in patients depending on long-term dialysis and patients with medically refractory SHPT.^[11]^ Remnant-conserving techniques for parathyroidectomy consist of subtotal parathyroidectomy (PTX), which removes all parathyroid tissue, leaving a small portion of a normal appearing gland in the neck, and total PTX with forearm autotransplantation (FAT), which can be helpful in removing all parathyroid tissue and also implanting small pieces of tissue at a distance in well-vascularized muscular structures.^[12–14]^ Although both techniques are now under criticism due to the high recurrence rate of SHPT, they still remain the most preferred and widely performed surgical procedures.^[1]^ Recently, total PTX without autotransplantation is regarded as a reliable surgical solution for patients suffering from severe SHPT, especially patients who have low perspective for renal transplantation.^[15]^ Herein, the study aims to explore the role of total PTX combined with FAT in the quality of life and recurrence of SHPT in chronic kidney disease patients by comparing the use of total PTX + FAT and total individual PTX.

## 2 Methods

### 2.1 Ethics statement

The study was approved by the Ethics Committee of Yinzhou Hospital of Ningbo University Medical College and written informed consents were obtained from all patients.

### 2.2 Study subjects

From January 2010 to May 2014, a total of 104 uremic patients suffering from SHPT diagnosed and treated in the Yinzhou Hospital of Ningbo University Medical College were divided into PTX groups (n = 62; patients underwent total PTX operation) and PTX + FAT groups (n = 42; patients underwent combined total PTX and FAT operation). Diagnosis criteria were as below: Nuclide scanning showed 1 or more hot nodules with highly concentrated 131I, and low iodine intake capability in thyroid tissue other than nodules was evaluated; size of the hot nodules was inspected by ultrasonography or computerized tomography scan to confirm the nodular goiter; clinical manifestations presented decreased arrhythmia, limb numbness, and limb proximal muscle strength; serum 25-hydroxyvitamin D (25OHD) < 30 ng/mL or estimated glomerular filtration rate (eGFR) < 60 mL/min/1.73 m^2^.^[16,17]^ The patients were included into this study following the criteria: patients were with good adherence for systematic treatment and observations; and patients were diagnosed with SHPT by preoperative clinical symptom, signs, and biochemical and X ray manifestations (patients were included into SHPT patients group when^[1]^ patients having intact PTH (iPTH) levels of >600 pg/mL accompanied with obvious calcium and phosphorus metabolic disorders^[2]^; patients unresponsive to available medical therapy^[3]^; patients suffering from severe osteoporosis and skeletal deformity observed by radiological examination^[4]^; patients showing severe clinical symptoms, such as bone pain, cutaneous pruritus, and coronary artery calcification (CAC). Patients with the following symptoms were excluded from the study: patients suffering from congenital, genetic, autoimmune, or cardiovascular diseases; patients with a history of heavy smoking, alcohol abuse, drug abuse, psychiatric problems, and patients in pregnant or suckling period; patients suffering from severe anemia, bleeding, coagulation dysfunction, and patients unsuitable for general anesthesia; patients having deformities in neck bone or soft tissues and patients suffering from morbid obesity; patients underwent neck operation, radiological treatment, and with hypertrophic scar; patients who had large thyroid nodules, thyroiditis, substernal ectopic parathyroid, and parathyroid carcinoma patients; patients who had a high PTH >35 pmoL/L and serum calcium >2.60 mmoL/L^[18]^; and patients who had bone hunger syndrome. All study subjects showed no obvious side reaction after heparin dialysis.

### 2.3 Operative procedures

Before the operation, the cardiopulmonary function of patients was estimated by routine blood examinations, blood biochemistry, X-ray chest radiographs, electrocardiographs (ECG), and cardiopulmonary function tests, aiming to assess patient tolerance for the operation. Adequate dialysis and strict dry weight control were confirmed to ensure a cardiothoracic ratio of < 60%. Hemodialysis was performed a day before the operation using low molecular weight heparin anticoagulatant (Sanofi-Aventis, Paris, France). B ultrasound positioning was adapted to reveal the exact location of parathyroid glands and its correlation with other organs around it. The operation procedures were as follows: marker pen, 0.9% NaCl ice cube, hammer, steel ruler, small Warburg retractor, microneedle holder, sterile thermos flask, balance-type scale, 5-0 suture, and self-made small instruments of table pad were prepared.

Totally, 62 SHPT patients underwent total PTX. Patients were general anesthetized and laid supinely. Then, a cervical collar incision (6 ∼ 8 cm) was made in the anterior muscles in the middle of the neck to fully expose the thyroid. Later, 4 parathyroid glands were entirely removed after they were identified around the thyroid gland and breast bone. Particular attention was paid in order to protect the recurrent laryngeal nerves during the above procedures. A combined PTX and FAT operation was performed on 42 SHPT patients. During this operation, the parathyroid glands were resected, sent for pathological examination. After confirmed to be diffuse hyperpolarized, the diffuse hyperpolarized parts were frozen, sliced into 1∼2 mm^3^ sections. After that, 20∼30 slices were separately incubated into the patients’ forearm muscles without arteriovenous and with fistula. The sarcolemma was sutured with a nonabsorbable suture (Johnson & Johnson Medical Equipment Co., Ltd., Shanghai, China).

Serum calcium, phosphorus, and alkaline phosphatase (ALP) levels were monitored dynamically and reviewed once a day from the first week to the third week after operation. If serum calcium level was less than (<) 1.8 mmoL/L or convulsions ensued, calcium gluconate (1 g; Wyeth Pharmaceuticals, Inc., Philadelphia, PA) would be intravenously injected to patients with active vitamin D_3_ (Wyeth Pharmaceuticals, Inc., Philadelphia, PA) orally taken. At 1 ∼ 2 days after the operation, half or total heparin anticoagulation hemodialysis was performed to patients according to the wound condition. Vital signs, incision bleeding, and drainage tube were carefully monitored. Close attention was paid to the vascular care to prevent the occurrence of phlebitis.

### 2.4 Efficacy evaluation

Venous blood of all subjects after 12 hours of fasting was collected in the morning before operation, and at 1 week, 1, 3, 6, and 12 months after operation, and routine blood tests were detected by the XE-2100 type automatic blood cell analyzer. Moreover, serum calcium, phosphorus, and alkaline phosphatase (ALP) levels were detected by the Hitachi-7600 automatic biochemical analyzer and the calcium-phosphorus product was calculated. Finally, iPTH levels were detected using chemiluminescent enzyme immunoassay. The signs, symptoms, and the MOS 36-item short-form health survey (SF-36) score, complication, and recurrence condition were recorded. An iPTH level > 300 pg/mL 12 months after operation was defined as recurrence.^[19]^ CAC was evaluated by the calcification in patients after the operation.^[20]^ The SF-36^[21]^ questionnaire was divided into the following 8 dimensions: physical functioning, mental health, social activity, emotional health, physical influence, vitality, physical pain, and general health to measure the quality of life. Accumulation method was applied to score each dimension and the lower score represented a lower quality of life.

### 2.5 Follow-up

A 12-month follow-up was conducted for all patients once every month. General information of patients, blood biochemical indicators, symptoms, remission of signs, complications, and recurrences were recorded and the SF-36 questionnaire was conducted. The follow-up ended by May 2015.

### 2.6 Statistical analysis

G∗Power3.1 was used to estimate the sample size. The previous study showed that the overall numerical rating scale (NRS) standard deviation was 2. NRS score difference of 1 represented a practical significance, and a = 0.05 revealed 80% efficiency with a total sample size of 95 cases or more. Data analyses were performed using the SPSS 20.0 (IBM Corp., Armonk, NY). Measurement data were expressed as mean ± standard deviation (SD). *T* test was adapted for the comparison between 2 groups, and repeated measurement analysis of variance (ANOVA) for comparison between 2 groups at different time points. Count data were expressed as percentage or rate, and the Chi-square test was used to compare the data between groups. The risk factors for the recurrences of the uremic patients with SHPT were analyzed by binary logistic regression analysis. *P* < .05 signifies a statistical difference.

## 3 Results

### 3.1 Baseline characteristics between the PTX and PTX + FAT groups

As presented in Table 1, gender and age of each group were not statistically significant (both *P* *>* .05). There was no statistical significance in dialysis type, dialysis age, primary disease, symptoms and signs, fracture site and blood biochemical indicators (iPTH, serum calcium and phosphorus, calcium-phosphorus product, and ALP), and SF-36 score between the PTX and PTX + FAT groups (all *P* *>* .05).

**Table 1:**
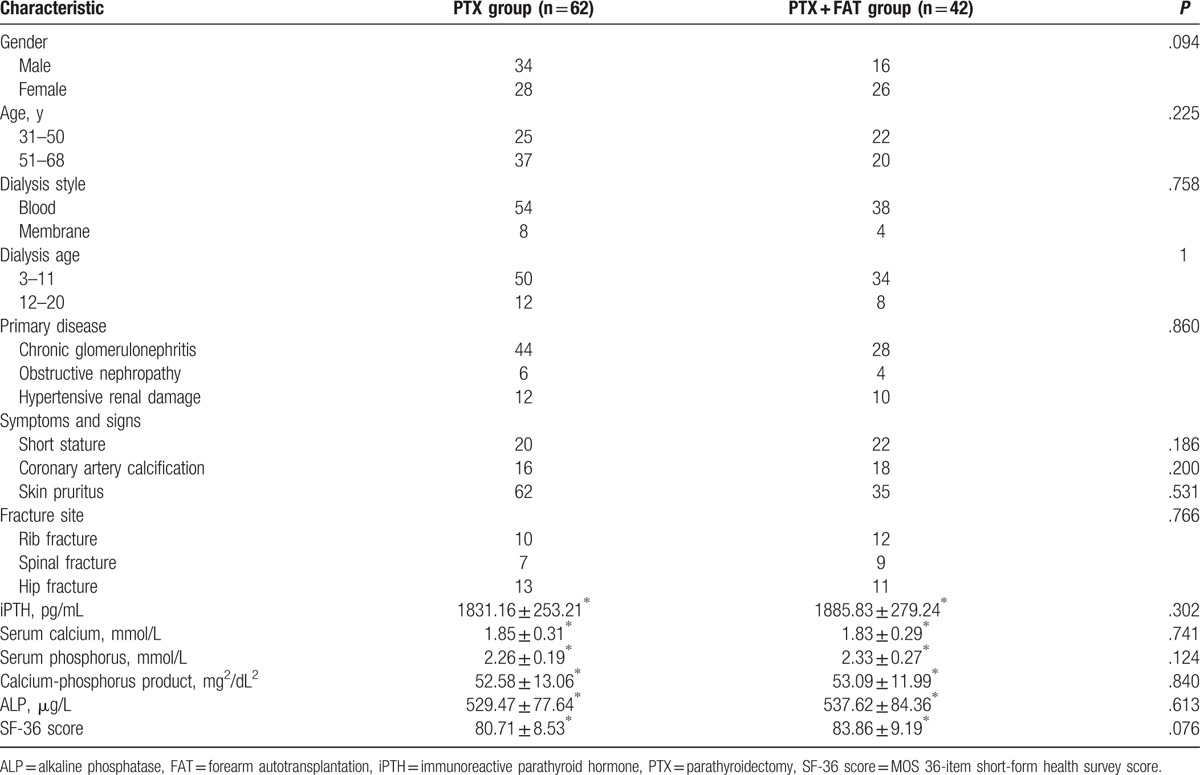
Comparisons of baseline characteristics between the PTX and PTX + FAT groups.

### 3.2 Blood biochemical indicators between the PTX and PTX + FAT groups

As summarized in Table 2, compared with those indicators before the operation, the PTX and PTX + FAT groups showed a decrease in postoperative levels of iPTH, serum phosphorus, serum calcium, and calcium-phosphorus product at all time periods after the operation (all *P* < .05), among which iPTH, serum phosphorus, and calcium-phosphorus product showed relatively small changes, and serum calcium levels initially decreased to the lowest level at the first week after the operation, and which subsequently increased thereafter. The blood biochemical indicators had no significant difference before the operation and 1 week after the operation (all *P* *>* .05). Compared with the PTX group, the iPTH levels decreased significantly, but serum calcium levels and the calcium-phosphorus product were significantly elevated in the PTX + FAT group at 3, 6, and 12 months after the operation (all *P* < .05). ALP levels increased and then declined in the PTX group. One week after the operation, the PTX and PTX + FAT groups showed a significant decreased ALP levels, and moreover, the PTX + FAT group showed a more prominently decreased ALP levels (all *P* < .05). The serum phosphorus levels showed no remarkable difference 1 week after the operation.

**Table 2:**
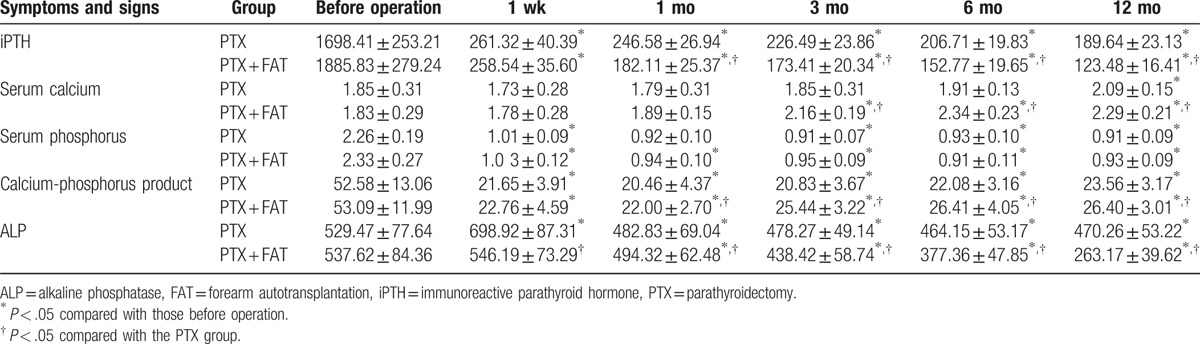
Comparisons of iPTH, serum calcium, serum phosphorus, and calcium-phosphorus product between the PTX and PTX + FAT groups.

### 3.3 Postoperative symptoms and signs between the PTX and PTX + FAT groups

The signs and symptoms of each group are summarized in Table 3. Compared with preoperative values, the bone pain and skin pruritus in the PTX and PTX + FAT groups decreased significantly at each time period (all *P* < .05); fractures or deformities and CAC decreased significantly upon measurement at 1, 3, 6, and 12 months after the operation (all *P* < .05). Short stature decreased significantly at 6 and 12 months after the operation in the PTX group, while it decreased significantly at 3, 6, and 12 months after the operation in the PTX + FAT group (all *P* < .05). Compared with the PTX group, the bone pain and skin pruritus in the PTX + FAT group showed no significant differences at any given time periods (all *P* > .05), and the differences among fractures, deformities, or short stature between the 2 groups were not statistically significant at the 1 week, and at 1 and 3 months (all *P* > .05). Fractures, deformities, and short stature in the PTX + FAT group decreased significantly at 6 and 12 months compared with the PTX group (all *P* < .05). Differences in CAC between the PTX and PTX + FAT groups were not statistically significant at 1 week, and at 1, 3, and 12 months. However, CAC decreased significantly at 6 months after the operation in the PTX + FAT group (*P* > .05).

**Table 3:**
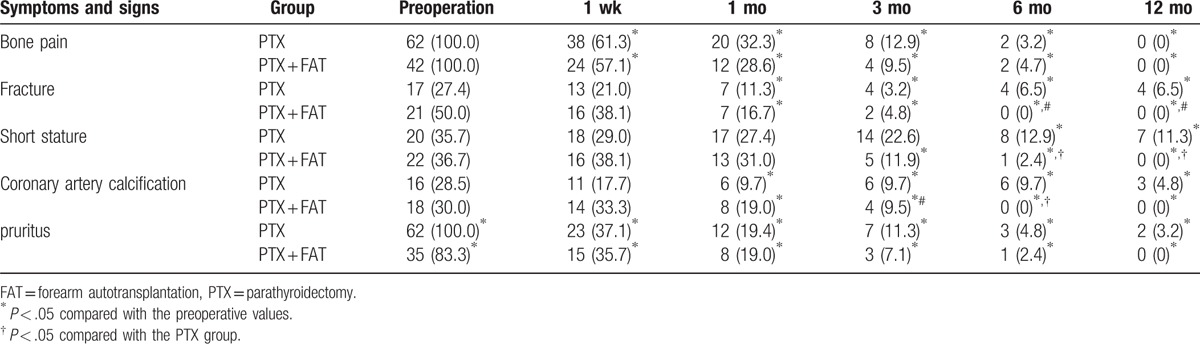
Comparisons of the operative symptoms and signs between the PTX and PTX + FAT groups [n (%)].

### 3.4 Comparison of SF-36 scores between the PTX and PTX + FAT groups

**Figure 1:**
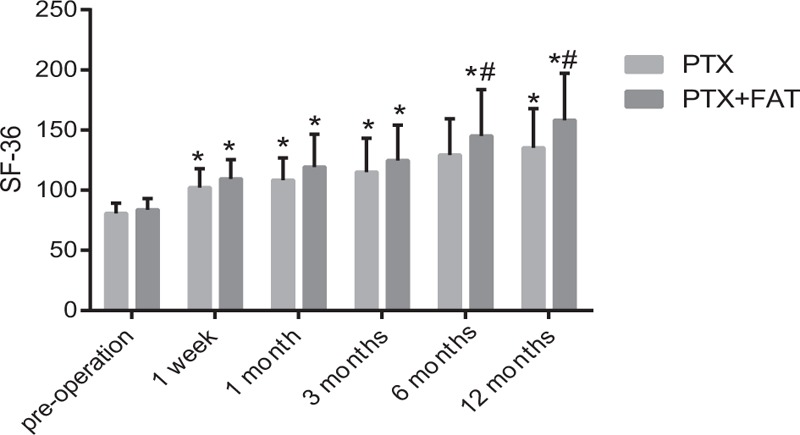
Comparison of SF-36 score in the PTX and PTX + FAT groups. Note: ^#^*P* < .05, compared with the PTX group. FAT = forearm autotransplantation, PTX = parathyroidectomy, SF-36 score = MOS 36-item short-form health survey score.

SF-36 scores in 3 groups are shown in Fig. 1. Compared with preoperative values, the SF-36 scores in the PTX and PTX + FAT groups increased significantly at different time periods after the operation (all *P* < .05). Compared with the PTX group, the SF-36 scores in the PTX + FAT group were not statistically significant at 1 week, and at 1 and 3 months (all *P* > .05), but the SF-36 scores increased significantly at 6 and 12 months (all *P* < .05).

### 3.5 Complication and recurrence between the PTX and PTX + FAT groups

**Figure 2:**
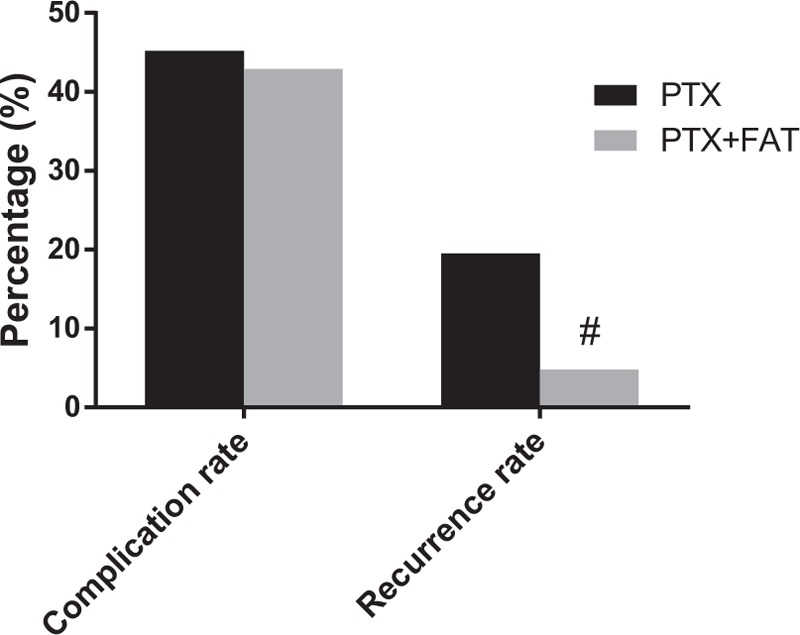
Comparison of complication and recurrence rate in the PTX and PTX + FAT groups. Note: ^#^*P* < .05, compared with the PTX group. FAT = forearm autotransplantation, PTX = parathyroidectomy.

Postoperative complications and recurrences 12 months after the operation are shown in Fig. 2. Compared with the PTX group, the complications in the PTX + FAT group were not statistically significant (*P* > .05), but the recurrence decreased significantly (*P* < .05). A total of 14 patients in the PTX group had recurrent laryngeal nerve injury (13 cases of unilateral recurrent laryngeal nerve injury and 1 case of bilateral recurrent laryngeal nerve injury), who slowly recovered within 7 to 10 days; 6 patients had severe low calcium convulsion, and 8 had pulmonary infection during the perioperative period. In the PTX + FAT group, 6 patients had blockage in internal fistula due to hypotension; 8 patients had unilateral recurrent laryngeal nerve injury, and 4 had severe low calcium convulsions.

### 3.6 Logistic regression analysis of risk factors for the recurrence of uremic patients with SHPT

Logistic regression analysis of recurrence-related factors for chronic kidney disease patients with SHPT is summarized in Table 4. Logistic regression analysis was conducted with the recurrence of patients being the dependent variable, and age, gender, dialysis type, dialysis age, preoperative iPTH level, preoperative SF-36 score, and operation methods of patients as independent variables. The results exhibited that preoperative iPTH levels (high level), SF-36 score (low score), and operation methods (PTX VS PTX + FAT) were the risk factors for the recurrence of uremic patients suffering from SHPT after operation (all *P* < .05). Also, the results showed that the patients treated with PTX individually had a higher recurrence rate of SHPT than patients treated by PTX + FAT. Moreover, an increased preoperative iPTH and a decreased preoperative SF-36 score signified an increased recurrence rate of SHPT.

**Table 4:**
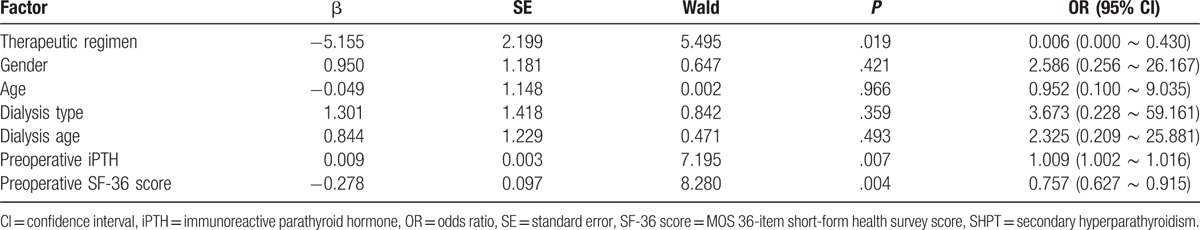
Logistic regression analysis of risk factors of the recurrence of uremic patients with SHPT.

## 4 Discussion

With the increasing number of patients suffering from SHPT, the need for parathyroidectomy increases as well. The search for better treatments for SHPT is imperative. Ogg,^[22]^ an American surgeon, in 1967 reported that PTX was proved to be an effective treatment for SHPT, although the patients suffered severe hypocalcemia and metabolic bone disease after the operation. It is reported that total PTX + FAT procedures had a high success rate and low recurrence rate, which was thus considered as the classic surgical procedure in the treatment of SHPT.^[23]^ Our study revealed the advantages of total PTX + FAT procedures in terms of postoperative life quality and recurrence of uremic patients with SHPT.

It was found that no deaths occurred among patients suffering from SHPT during the perioperative period of PTX or PTX + FAT procedures. It is reported that total PTX without autotransplantation could be the viable choice for treating patients with SHPT with a relative low recurrent rate and short operative time.^[24]^ He et al^[13]^ also demonstrated that total PTX + FAT procedure was a safe, feasible, and effective surgical option for SHPT patients owing to the results that reveal no deaths occurred among the 47 study subjects. The results of SF-36 questionnaire indicated that the quality of life in the PTX and PTX + FAT groups significantly increased after the operation compared with those preoperatively. Moreover, the SF-36 score at 6 and 12-month time periods after the operation in the PTX + FAT group was very close to that in the control group. From the results, we can say that total PTX and PTX + FAT procedures are effective in promoting the quality of life of patients suffering from SHPT, but PTX + FAT is far more advanced and effective than PTX. Rao et al^[25]^ demonstrated in their study that among the 283 patients suffering from mild asymptomatic primary hyperparathyroidism, 53 of them chose PTX, whereas the rest chose to go with the conservative treatment, and the SF-36 score in the 53 patients was similar to the normal population. In a previous study, the risk of reaching overly low iPTH value appears to increase after total PTX, which predisposes the patient to adynamic bone disease, increasing the risk of fractures and lowering the patient's quality of life.^[20]^

Besides, the levels of serum calcium, calcium-phosphorus product increased; the ALP, fracture or deformity, short stature; and the CAC decreased in the PTX + FAT group. According to the former study, patients were diagnosed to be hypoparathyroidism with a low serum calcium level, and the intravenous calcium levels were required for patients from performed the PTX + AT operation,^[26]^ which conforms to increased level of serum calcium in patients after PTX + FAT operation. Jing et al^[14]^ found that calcitriol was in need for postoperative patients suffering from uremia SHPT, and also the muscle weakness and bone pain muscle weakness were much relieved in patients after PTX + AT operation. It was revealed by previous research that the serum bone-specific ALP level presented the bone turnover and the bone scintigraphy was seen high in patients with SHPT,^[27]^ thus making the decreased ALP meaningful for patients after PTX + FAT operation. An improved bone fracture, bone pain, and tendon rupture were demonstrated in patients after PTX + AT operation.^[28,29]^ As been revealed by former evidence, the calcium levels of patients after operation were lower in the PTX operation group, while higher in the PTX + AT group.^[26]^ A lower CAC score was detected in patients after PTX + AT operation.^[20]^ From all the change of biological plausibility occurred to patients after PTX + AT operation, the advantage of PTX combined with FAT was shown obviously.

Most importantly, compared with the PTX group, the recurrence in the PTX + FAT group decreased significantly. As shown in a previous study, the recurrence rates in patients with SHPT were lower after total PTX + FAT than individual PTX.^[26]^ Recurrence may be associated with residual parathyroid tissue, hyperplasia of transplanted parathyroid tissue, or ectopic parathyroid.^[30]^ Although ultrasound and radionuclide scanning technology have matured a lot over the last few years, the failure in locating the entire parathyroid tissue still remains.^[31]^ Tissue traction often results in parathyroid ectopic.^[32]^ Therefore, preoperative parathyroid localization is imperative. The PTX group had 14 cases of recurrent laryngeal nerve injury (13 cases of unilateral recurrent laryngeal nerve injury and 1 case of bilateral recurrent laryngeal nerve injury), 6 cases of severe low calcium convulsion, and 8 cases of pulmonary infection during perioperative period. In the PTX + FAT group, 6 cases of blockage in internal fistula due to hypotension, 8 cases of unilateral recurrent laryngeal nerve injury, and 4 cases of severe low calcium convulsions were observed. Recurrent laryngeal nerve injury may be associated with intraoperative traction of recurrent laryngeal nerve, and therefore, the requirement to entirely expose the recurrent laryngeal nerve is necessary and it is not only favorable for the progression of the operation, preventing recurrent laryngeal nerve injury, but also beneficial for the resection of parathyroid and adipose tissue around lymph node.^[33]^ Hypocalcemia was supposed to be associated with the decreased secretion of vitamin D. It is reported that the secretion of 1, 25 (OH) 2D3 (a member of vitamin D) was significantly decreased in patients with renal failure, resulting in hypocalcemia.^[14]^ A majority of the patients felt that their symptoms were significantly relieved after the operations in both groups. Patients with bone pain and skin pruritus revealed obvious improvements in both groups. A previous study demonstrated significant association between serum calcium level and skin pruritus, and in our study, it was suggested to be related to decreased calcium levels after the operation.^[34]^ Fractures or deformities, extraskeletal calcification, and short stature decreased significantly in both groups. Moreover, patients with the aforementioned symptoms recovered faster and more efficiently in the PTX + FAT group than the patients in the PTX group. In addition, we discovered that serum phosphorus levels decreased significantly and maintained a relatively low level in both groups. Dhingra et al^[35]^ reported that a higher serum phosphorus level is associated with an increased risk of chronic kidney disease. The results in our study showed that the iPTH levels decreased significantly but persisted, and there was no significant difference in the postoperative values of each group, suggesting it would not cause loss of iPTH level.^[11]^

To conclude, our study proves that PTX + FAT procedures are comparatively safer and more effective in the treatment for uremic patients suffering from SHPT owing to its advantages in improving the quality of life and inhibiting the recurrence of the patients. However, the sample size of our study was too small in order to determine the most appropriate approach for SHPT and the follow-up period was only a short span of 12 months; therefore, further studies with larger sample size and longer follow-up durations are needed to support the findings.

## Acknowledgment

The authors acknowledge all the reviewers who had given supports for our article.
